# Detection of *Leptospira interrogans* DNA in Urine of a Captive Ocelot (*Leopardus pardalis*)

**DOI:** 10.3390/ijerph18020793

**Published:** 2021-01-19

**Authors:** Lucas N. Paz, Camila Hamond, Melissa H. Pinna

**Affiliations:** Bacterial Disease Laboratory (LABAC), School of Veterinary Medicine and Zootechny, Federal University of Bahia, Adhemar de Barros Avenue, 500, Salvador 40170-110, Brazil; lucasnpaz@hotmail.com (L.N.P.); camilahamond@gmail.com (C.H.)

Dear editor,

We read with interest the article recently published by Murillo and collaborators (2020) [1] in The *International Journal of Environmental Research and Public Health*. The main objective of the study was to evaluate the presence of antibodies against pathogenic *Leptospira* species and the prevalence of pathogenic *Leptospira* DNA in the urine and blood in stray cats in Spain. According to the authors, 4.1% of the animals were seropositive for *Leptospira* spp. (cutoff = title of 20). One sample (1.12%) was positive for the detection of *Leptospira* DNA. It is noteworthy that this cat did not have antibodies against *Leptospira* detected by MAT.

Anti-*Leptospira* antibodies have been described in domestic cats [1,2,3] and free-living felines worldwide [4,5,6]. In Brazil, studies with different species of wild felines, free-living or captive, have shown the expose of leptospires in these animals, which vary from 2.5% to 18.2% [4,7,8]. These studies are notable for characterizing the circulation of *Leptospira* in a large neotropical felines species from different epidemiological settings. However, in order to characterize chronic carrier animals, it is necessary to investigate the presence of DNA or obtain leptospiral isolates from urine or kidney samples [9].

In recent years, studies have demonstrated the presence of leptospire DNA in the urine and blood of domestic cats [1,3]. Additionally, Alashraf and collaborators (2020) [10] describe, for the first time, the recovery of *Leptospira interrogans* from urine and kidney samples from naturally infected domestic cats. Such results demonstrate the possibility that domestic cats act as chronic carriers of *Leptospira* ssp. Studies that characterize wild animals, especially wild felines, as chronic carriers are rare in the literature. Here, we describe for the first time the presence of *Leptospira* spp. DNA recovered from the urine of a naturally infected captive ocelot (*Leopardus pardalis*) and without clinical symptoms of leptospirosis. This study was carried out as part of the routine surveillance conducted by the Park’s veterinary team.

The microscopic agglutination test (MAT) was performed as recommended by the World Organization for Animal Health [11], using a panel composed of 19 serogroups (Table S1). In MAT, reactivity was observed with the titer of 25 for serovar Canicola. For direct identification of the agent, the amplification was primarily directed towards the detection of the *lipL32* gene (present only in pathogenic *Leptospira* species). Then, the *secY* housekeeping gene (responsible for determining the *Leptospira* species) was amplified and the amplicons were purified and sequenced as described by Paz and collaborators (2019) [12]. Genotyping based on the partial *secY* gene characterized the infecting bacteria as *Leptospira interrogans* (GenBank accession number MW013523). Phylogenetic analysis was performed with Mega v6 software using the neighbor-joining method. The phylogenetic tree (Figure 1) was built using the Tamura–Nei model [13].

We observed that even in the presence of the bacteria, the anti-*Leptospira* antibody titer was low. These results corroborate with previous studies that demonstrate the presence of DNA [1] or the obtaining of isolates [10] from *Leptospira* spp. in urine and kidney samples in domestic cats with low or negative MAT titers. Murillo and collaborators (2020) [1] suggest that chronically infected cats can have stable, decreasing or not present antibody titers. In addition, these animals, that do not show antibody titers by MAT, can remain eliminating viable leptospires through urine and serve as a source of infection for other animals and humans.

The identification of wild species as reservoirs of *Leptospira* brings relevant information about the epidemiology of this pathogen [14]. Domestic cats naturally infected are able to release the pathogenic DNA of leptospira in their urine for up to eight months [15]. Salvador has regions with high rates (37.8 per 1000 individuals per year) of infection for human leptospirosis [16,17]. In addition, pathogenic Leptospira has recently described in surface waters from the urban environment [18]. We infer that these animals may act as chronic carriers of *L. interrogans* and be a possible source for transmission of the bacteria to other wild and domestic animals or humans, especially for other captive animals and for zoo staff. Although the epidemiological importance of these animals in the leptospirosis transmission chain is not known, our data can assist in the surveillance of leptospirosis, as well as suggest a possible risk to animal and human health and point out new directions for future studies.

## Figures and Tables

**Figure 1:**
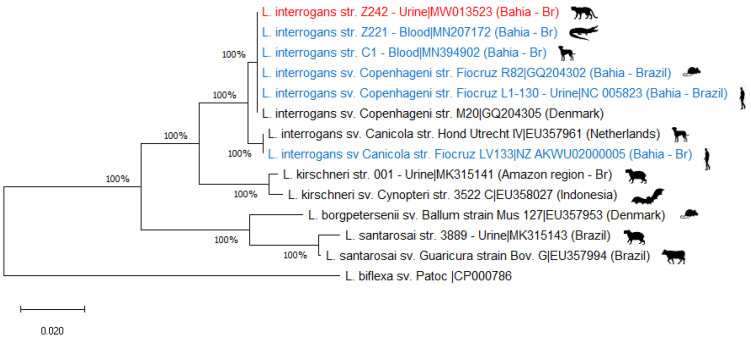
Phylogenetic tree built with *secY* gene sequence analysis recovered from blood sample of an Ocelot (*Leopardus pardalis*). Sequence obtained directly from clinical samples (urine) studied in this work are indicated in red, sequences obtained from a *L. interrogans* strain from, dog, broad-Snouted Caiman, rodents isolated and human, in the same regions are in blue. Additional sequences corresponding to isolates obtained elsewhere from a variety of hosts are indicated in black. After the vertical bar, the GenBank accession number is reported for each sequence. The analysis was made using the maximum-likelihood method (Tamura–Nei model). The support for the branching order was determined by 1000 bootstrap.

## Supplementary Materials

The following are available online at https://www.mdpi.com/1660-4601/18/2/793/s1, Table S1: Leptospiral antigens used on MAT.
