# Supplementary material for: Detection of Leptospira interrogans DNA in Urine of a Captive Ocelot (Leopardus pardalis)
Source: Int J Environ Res Public Health. 2021 Jan 19;18(2):793. doi: 10.3390/ijerph18020793 (PMC7832400; doi:10.3390/ijerph18020793)
Supplement: Supplementary file 1 [file ijerph-18-00793-s001.pdf]

Table S1. Leptospiral antigens used on MAT.

**Species**	**Serogroup**	**Serovar**	**Strain**
L. interrogans	Australis	Australis	Ballico
L. interrogans	Autumnalis	Autumnalis	Akiyami A
L. borgpetersenii	Ballum	Ballum	Mus 127
L. interrogans	Bataviae	Bataviae	Swart
L. interrogans	Canicola	Canicola	Hond Utrecht IV
L. weilli	Celledoni	Celledoni	Celledoni
L. kirschneri	Cynopteri	Cynopteri	3522C
L. interrogans	Djasiman	Djasiman	Djasiman
L. Kirschneri	Grippotyphosa	Grippotyphosa	Duyster
L. interrogans	Hebdomadis	Hebdomadis	Hebdomadis
L. interrogans	Hebdomadis	Kremastos	Kremastos
L. interrogans	Icterohaemorrhagiae	Copenhageni	M20
L. interrogans	Icterohaemorrhagiae	Copenhageni	L1 130
L. weilli	Javanica	Coxi	Cox
L. borgpetersenii	Javanica	Poi	Poi
L. noguchii	Lousiana	Lousiana	1945
L. noguchii	Panama	Panama	CZ 214 Z
L. interrogans	Pomona	Pomona	Pomona
L. interrogans	Pyrogenes	Pyrogenes	Salinem
L. interrogans	Sejroe	Hardjo	Hardjoprajitno
L. borgpetersenii	Tarassovi	Tarassovi	Perepelicin
